# Tandem mass tag labeled quantitative proteomic analysis of differential protein expression on total alkaloid of *Aconitum flavum* Hand.-Mazz. against *melophagus ovinus*

**DOI:** 10.3389/fvets.2022.951058

**Published:** 2022-07-27

**Authors:** Xinjian Wang, Zhen Yang, Yujun Zhang, Feng Cheng, Xiaoyong Xing, Fengqin Wen, Yonghao Hu, Changjiang Chen, Bin Wei, Pengxia Bai, Xuehong Wang, Yu Liu, Hongjuan Zhang, Baocheng Hao, Shengyi Wang

**Affiliations:** ^1^Key Laboratory of New Animal Drug Project, Gansu Province, Key Laboratory of Veterinary Pharmaceutical Development, Ministry of Agriculture and Rural Affairs, Lanzhou Institute of Husbandry and Pharmaceutical Sciences of Chinese Academy of Agriculture Sciences, Lanzhou, China; ^2^College of Veterinary Medicine, Gansu Agricultural University, Lanzhou, China; ^3^Animal Husbandry and Veterinary Station of Huangyuan County, Xining, China; ^4^Qinghai College of Animal Husbandry and Veterinary Technology, Xining, China

**Keywords:** *melophagus ovinus*, TMT labeled quantitative proteomics, killing activity, mechanism, total alkaloids

## Abstract

*Melophagus ovinus* disease is a common ectoparasitosis, which can lead to a decrease in animal production performance, product quality, and even death. *Aconitum flavum* Hand.-Mazz. has many pharmacological activities including insecticidal, heat-clearing, analgesic, and dehumidifying. However, there are few researches focused on the effects and related mechanism of *Aconitum flavum* Hand.-Mazz. in killing *Melophagus ovinus*. In this study, 11 alkaloids of *Aconitum flavum* Hand.-Mazz. were detected, and its total alkaloid activity was determined. The results showed when the total alkaloid concentration was 64 mg/ml and the treatment time was 16 h, the killing rate of *Melophagus ovinus* reached 100%. Through the observation of the differences in the surface of *Melophagus ovinus* in each experimental group, it was found that the morphology of the posterior end of the female *Melophagus ovinus* in the alkaloid treatment group was significantly different from that of the blank and positive control groups, and most of the epidermal tissue was obsessive and missing. Moreover, the enzyme activity determination results of 64 mg/ml group were significantly different when compared with the normal control group, while there was no significant difference in other groups. Then, the *Melophagus ovinus* gene library was established by the unreferenced genome transcriptome sequencing, the proteomic comparison was performed using tandem mass tag labeled protein detection technology, and finally, the samples were quantitatively analyzed by liquid chromatography-mass spectrometry tandem and bioinformatics methods. Based on the above experimental results, it was speculated that *Aconitum flavum* Hand.-Mazz. total alkaloids may cause the imbalance of protein disulfide isomerase expressions by affecting the regulation of Hsp40 cellular protein homeostasis and the oxidation of protein disulfide isomerase and related proteins. This would affect the selective recognition of signal sequence, the targeted transport of Sec 61, and the correct folding of the three-dimensional structure of amino acid chain, weakening the clearance of amino acid chains that cannot be correctly folded and eventually resulting in the killing of *Melophagus ovinus*. This study preliminarily revealed the mechanism of *Aconitum flavum* Hand.-Mazz. total alkaloids against *Melophagus ovinus* and provided a theoretical basis for the screening of *Melophagus ovinus* action targets and the development of new veterinary drugs.

## Introduction

*Melophagus ovinus* disease is a common external parasitic disease caused by blood-sucking *Melophagus ovinus* parasitic on animal surfaces, which is characterized by itchy skin, chronic inflammation, and direct contact transmission. It mainly occurs in sheep, dogs, horses, etc., resulting in animal emaciation, anemia, poor growth, decreased quality of livestock products, and even death of individual animal pups. Besides, the most serious harm of *Melophagus ovinus* is that it can be used as a vector to spread bluetongue virus (1), *Trypanosoma* (2), *Bartonella* (3), *Rickettsia* (4), *Acinetobacter* (5), and other pathogens. Among them, bluetongue is one of the most susceptible sheep infectious diseases, with an incidence rate of 76% (6), causing great harm to animal husbandry.

*Melophagus ovinus* are widely distributed in Australia, Mongolia, India, Japan, and other places. The prevalence of this parasite has also been reported in Qinghai, Xinjiang, Shandong, and other provinces of China (7). At present, permethrin, phoxim, ivermectin, and other antibiotics and chemical drugs are the main ways to control the disease, while the immune intervention and biological control are still in the research stage (8). Although antibiotics and chemical insecticides have the advantage of high efficacy, rapidity, and convenience, drug residues and drug resistance caused by their long-term large-scale use will pose a serious threat to food safety and public health, and this problem has attracted the strong worldwide attention (9, 10).

With the enhancement of people's awareness of health and environmental protection, natural plant insecticides have attracted considerable attention due to their low drug resistance, low residue, and relative safety to human, livestock, and environment (11, 12). It is reported that the application of plant-derived drugs can significantly reduce the animal medicine costs of farms. For example, the average medicine cost per farm in the Netherlands dropped to 5,500 euros in 2009, which was the lowest in 20 years (13). Therefore, as a research hotspot in recent years, finding safe and efficient insecticides from medicinal plants has broad application prospects and important social value (14).

*Aconitum flavum* Hand.-Mazz. (here after referred to as AFHM) belongs to the genus *Aconitum* in the family of Ranunculaceae. It mainly grows in the mountainous grassy slopes or sparse forests with an altitude of 2,000–3,700 m in Tibet, Gansu, Qinghai, Shanxi, Ningxia, Sichuan, and other provinces of China and is used as medicinal materials in these areas. According to modern pharmaceutical research, the main active components of AFHM are diester diterpenoid alkaloids. Zhang et al. used electrospray ionization tandem mass spectrometry and high-resolution electrospray ionization mass spectrometry to analyze the total alkaloids in AFHM roots and detected five main alkaloids including 12-epi-napelline, 3-deoxyaconitine, aconitine, 3-deoxyaconitine-8-linoleate, and aconitine-8-linoleate (15). *Aconitum* plants have been reported to have certain neurotoxicity and cardiotoxicity when taken orally (16, 17), but more reports pointed out that it has a wide range of pharmacological activities (18–21), such as anti-inflammatory, analgesic, rheumatism, insecticidal, and anti-tumor.

At present, the research on the mechanism of drug prevention and treatment of animal parasitic diseases is mainly reflected indirectly by the evaluation of the activities of metabolic enzymes in animal blood. For example, Shang et al. (22) measured the activity of antioxidant enzymes in *Psoroptes-*infested rabbits. The results showed that the SOD activity in the infection group was weakly inhibited, but not significantly different from the blank group, while the activities of GST, CAT, and MDA were significantly increased (*P* < 0.01). In addition, the molecular levels of GSTs, ABC transporters, sodium ion voltage-gated channels, and pH-gated chloride channels have also been reported to be related to the mechanisms of action or resistance of ivermectin and other drugs (23, 24). However, there are few reports on the effect of drugs on enzyme activity of *Melophagus ovinus* and their killing mechanism at the protein level.

Protein is the main embodiment of biological function which controls and regulates many life activities through its own unique activities. TMT labeling technology, which is more sensitive and accurate than 2-DE and MS, was utilized in this study for proteomic analysis, aiming to determine the effect of total AFHM alkaloids on the enzymatic activity and differential protein expression of *Melophagus ovinus*. This study provided a theoretical basis for screening the action targets of *Melophagus ovinus* and developing new veterinary drugs.

## Materials and methods

### Materials and reagents

The roots of AFHM were purchased from Gansu Fuxinghou Biomedical Technology Co., Ltd. The specimen of AFHM (20200311) was kept in the Key Laboratory of Veterinary Pharmaceutical Development of the Ministry of Agriculture, CAAS Lanzhou Institute of Husbandry and Pharmaceutical. The *Melophagus ovinus* were collected from sheep farms in Huangyuan County, Qinghai Province. Anhydrous ethanol was purchased from Sinopharm Chemical Reagent Co., Ltd.; petroleum ether and chloroform were purchased from Tianjin Damao Chemical Reagent Factory; ammonia water was brought from Yantai Shuangshuang Chemical Co., Ltd. Methanol, acetonitrile, and isopropanol used in experiments were chromatographic grade and obtained from Fisher Chemical. The standard aconitine (production batch number: DST200330-006) and 3-deoxyaconitrile (production batch number: DST200810-033) were purchased from Lemeitian Pharmaceutical/Desit Biological. Phosphate buffered saline (PBS), BCA protein concentration determination kit, catalase (CAT), peroxidase (POD), carboxylesterase (CarE), glutathione peroxidase (GSH-Px), monoamine oxidase (MAO), acetylcholinesterase (AChE), and Ca^2+^-Mg^2+^-ATPase detection kits were purchased from Solarbio Science & Technology Co., Ltd. (Beijing, China).

### Preparation and preliminary analysis of AFHM total alkaloid extract

The dry powder of AFHM was soaked in 95% ethanol for 24 h (mass/volume ratio was 1:10), and the extraction was repeated three times. The combined extract was concentrated under reduced pressure on a rotary evaporator at 60°C, 70 rpm/min, and 0.076 MPa to obtain the ethanol extract. Furthermore, the ethanol extract was dissolved in 2% HCl and centrifuged at 4,000 rpm for 10 min. The supernatant was filtrated twice and extracted with petroleum ether for three times. The lower aqueous solution was adjusted to pH 9.5 with alkali solution, and the obtained yellow precipitate was alkaloid A. The supernatant was extracted with equal volume chloroform for five times, and the lower layer of chloroform extract was collected and concentrated under reduced pressure to obtain the colloidal substance which was alkaloid B. The total alkaloid of AFHM was the mixture of A and B.

The mixture was dissolved with 3 ml of chromatographic methanol, and the sample composition was detected by HPLC/QTOF-MS with the specific detection conditions as follows: High Performance Liquid Agilent 1290 HPLC, Waters UPLC BEH C18 column (1.7 × 2.1 μm × 100 mm), 0.1% formic acid solution as mobile phase A, acetonitrile-0.1% formic acid solution as mobile phase B, 5 μl as the injection volume, and 400 μl/min as the flow rate. The total biological base of AFHM was dissolved and diluted to 3 mg/ml with chromatographic methanol and filtered with 0.22 μM filter before use. Standard aconitine and 3-deoxyaconitine were dissolved in chromatographic methanol to prepare 0.800 and 0.379 mg/ml solutions, respectively, and then filtered by a 0.22 μM filter. The samples were used for chromatographic detection with conditions of high-performance liquid chromatography Agilent 1290 HPLC, Agilent C18 column (4.6 × 250 mm, 5 μm), 0.2% phosphoric acid-0.4% triethylamine solution as mobile phase A, acetonitrile as mobile phase B, 10 μl as the injection volume, 0.8 ml/min as the flow rate and 240 nm as the detection wavelength.

### Killing effect of AFHM total alkaloids *in vitro*

The *Melophagus ovinus* with body lengths of 4–6 mm were randomly divided into seven groups: blank control group, positive control group (1% phoxim), and drug treatment groups (the total alkaloids concentrations of AFHM were 8, 16, 32, 64, and 128 mg/ml, respectively). Each group was set with three replicates, and there were 10 *Melophagus ovinus* in each replicate. The observation time points of mortality of *Melophagus ovinus* were set to 1, 2, 4, 8, 16, 24, 48, 72, 96, 120, and 136 h.

### Observation of body surface differences of *Melophagus ovinus* before and after drug treatment

Stereomicroscope and scanning electron microscope were used to observe the body surface differences of *Melophagus ovinus* before and after treatment with AFHM total alkaloids. *Melophagus ovinus* in each group were treated with cleaning, fixing, and gold spraying, and the differences of body appearance were observed under 35 × scanning electron microscope.

### Effects of AFHM total alkaloids on key enzyme activities of *melophagus ovinus*

The *Melophagus ovinus* with body lengths of 4–6 mm were randomly divided into seven groups as described in Section Killing effect of AFHM total alkaloids *in vitro*. The activities of catalase (CAT), peroxidase (POD), carboxylesterase (CarE), glutathione peroxidase (GSH-Px), monoamine oxidase (MAO), acetylcholinesterase (AChE), and Ca^2+^-Mg^2+^-ATPase in each group were determined by biochemical analysis kits after 16 h treatment (Solarbio Science & Technology Co. Ltd., China).

### Unreferenced genome transcriptome sequencing of *melophagus ovinus*

Thirty *Melophagus ovinus* used for unreferenced genome transcriptome sequencing were collected and randomly divided into three groups. Guanidine isothiocyanate-phenol-chloroform-one-step method was used to extract the total RNA of *Melophagus ovinus*. The mRNA with poly A structure in total RNA was enriched by oligo (DT) magnetic beads, and the RNA was interrupted to a fragment with a length of about 300 bp by ion interruption. Using RNA as a template, the first strand cDNA was synthesized with six-base random primers and reverse transcriptase, and the second strand cDNA was synthesized with the first strand cDNA as a template. After the library was constructed, the library fragments were enriched by PCR amplification. And, the library was selected according to the fragment size of 450 bp. The total and effective concentrations of the library were detected using an Agilent 2100 Bioanalyzer. Then, libraries containing different index sequences (each sample plus different indexes, and finally distinguishing the off-line data for each sample based on metrics) were mixed proportionally according to the effective concentration and the amount of data required. The mixed library was uniformly diluted to 2 nM, and alkali denatured to form a single chain library. After RNA extraction, purification, and library construction, these libraries were sequenced by next-generation sequencing (NGS) based on the Illumina HiSeq sequencing platform (Shanghai Personal Biotechnology Co., Ltd).

### Study on the mechanism of killing *melophagus ovinus* with AFHM total alkaloids based on differential proteomics

#### Protein extraction

The *Melophagus ovinus* were randomly divided into a blank control group and an AFHM group (64 mg/ml), with three replicates in each group and 10 *Melophagus ovinus* in each replicate. Samples were collected when the treatment time was 16 h. The sample was ground to cell powder in liquid nitrogen and transferred to a 5-ml centrifuge tube. Four volumes of lysis buffer (8 M urea, 1% protease inhibitor cocktail) were then added to the cell powder, and the mixture was sonicated three times on ice using a high-intensity ultrasonic processor (Scientz) (Note: For PTM experiments, inhibitors were also added to the lysis buffer, e.g., 3 μM TSA and 50 mM NAM for acetylation and 1% phosphatase inhibitor for phosphorylation). The remaining debris was removed by centrifugation at 12,000 g at 4°C for 10 min. Finally, the supernatant was collected, and the protein concentration was determined with the BCA kit according to the manufacturer's instructions.

#### Trypsin digestion

The protein solution was reduced with 5 mM dithiothreitol for 30 min at 56°C and then alkylated with 11 mM iodoacetamide for 15 min at room temperature in darkness. Next, the protein sample was diluted by adding 100 mM TEAB to a urea concentration <2 M. The first digestion was performed overnight at a 1:50 trypsin-to-protein mass ratio, and the second digestion was performed for 4 h at a 1:100 trypsin-to-protein mass ratio. Finally, the obtained peptides were desalted by the C18 SPE column.

#### TMT-labeling

The TMT-based LC-MS/MS identification and data normalization analysis were performed by PTM-biolab™ (Hangzhou, China) and Shanghai Personal Biotechnology Co., Ltd. Tryptic peptides were first dissolved in 0.5 M TEAB. Each channel of the peptide was labeled with its respective TMT reagent (based on manufacturer's protocol, ThermoFisher Scientific) and incubated for 2 h at room temperature. Five microliters of each sample were pooled, desalted, and analyzed by MS to check labeling efficiency. Then, 5% hydroxylamine was added to the samples for quenching. The pooled samples were then desalted with Strata X C18 SPE column (Phenomenex) and dried by vacuum centrifugation.

#### HPLC fractionation

The sample was fractionated into fractions by high pH reverse-phase HPLC using an Agilent 300 Extend C18 column (5 μm particles, 4.6 mm ID, 250 mm length). Briefly, peptides were separated into 80 fractions with a gradient of 2–60% acetonitrile in 10 mM ammonium bicarbonate (pH 10) over 80 min. Then, the peptides were combined into nine fractions and dried by vacuum centrifugation.

#### LC-MS/MS analysis

The tryptic peptides were dissolved in solvent A (0.1% formic acid, 2% acetonitrile in water) and directly loaded onto a homemade reversed-phase analytical column (25-cm length, 75 μm i.d.). Peptides were separated with different concentration gradients of solvent B (0.1% formic acid in 90% acetonitrile), i.e., a gradient of 5–25% for 60 min, a gradient of 25–35% for 22 min, climbing to 80% in 4 min, and holding for 4 min. All operations were performed at a constant flow rate of 450 nl/min on an EASY-nLC 1200 UPLC system (ThermoFisher Scientific).

The separated peptides were analyzed in Q ExactiveTM HF-X (ThermoFisher Scientific) with a nano-electrospray ion source. The electrospray voltage applied was 2.0 kV. The full MS scan resolution was set to 60,000 for a scan range of 3501,600 m/z. Up to 20 most abundant precursors were then selected for further MS/MS analyses with 30 s dynamic exclusion. The HCD fragmentation was performed at a normalized collision energy (NCE) of 28%. The fragments were detected in the Orbitrap at a resolution of 30,000. The fixed first mass was set as 100 m/z. The automatic gain control (AGC) target was set at 1E5, with an intensity threshold of 3.3E4 and a maximum injection time of 60 ms.

#### Database search

The resulting MS/MS data were processed using the MaxQuant search engine (v.1.6.15.0). Tandem mass spectra were searched against the SRA data (Accession ID: PRJNA836644) and UniProt Knowledgebase concatenated with reverse decoy database. Trypsin/P was specified as a cleavage enzyme allowing up to two missing cleavages. The mass tolerance for precursor ions was set as 20 ppm in first search and 5 ppm in main search, and the mass tolerance for fragment ions was set as 0.02 Da. Carbamidomethyl on Cys was specified as a fixed modification, and acetylation on protein N-terminal and oxidation on Met were specified as variable modifications. FDR was adjusted to <1%.

#### Bioinformatics analysis

##### GO annotation

The Gene Ontology, or GO, is a major bioinformatics initiative to unify the representation of genes and gene product attributes across all species. More specifically, the project aims to maintain and develop its controlled vocabulary of genes and gene product attributes, annotate genes and gene products, assimilate and disseminate annotation data, and provide tools to facilitate access to all aspects of the project data. The gene ontology covers three domains including cellular component, molecular function, and biological process. The cellular component is a component of a cell, but with the proviso that it is part of some larger object. This may be an anatomical structure (e.g., rough endoplasmic reticulum or nucleus) or a gene product group (e.g., ribosome, proteasome, or a protein dimer). Molecular function describes activities, such as catalytic or binding activities, that occur at the molecular level. GO molecular function terms represent activities rather than the entities (molecules or complexes) that perform the actions and do not specify where, when, or in which context the action takes place. The biological process is a series of events accomplished by an ordered assembly of one or more molecular functions. Biological processes and molecular functions are difficult to distinguish, but the general rule is that a process must have multiple distinct steps. Gene ontology (GO) annotation proteome was derived from the UniProt-GOA database (http://www.ebi.ac.uk/GOA/). First, the identified protein IDs were converted to UniProt IDs and then mapped to GO IDs by protein IDs. If some identified proteins were not annotated by the UniProt-GOA database, the InterProScan software would be used to the annotated protein's GO functional based on the protein sequence alignment method. Then, proteins were classified by Gene Ontology annotation based on three categories: biological process, cellular component, and molecular function.

##### Domain annotation

Protein domains are conserved parts of a specific protein sequence and structure that can evolve, function, and exist independently of the rest of the protein chain. Each domain forms a compact three-dimensional structure and can normally be stabilized and folded independently. Many proteins consist of several structural domains. One domain may appear in a variety of differentially expressed proteins. Molecular evolution uses domains as building blocks that can be recombined in different arrangements to create proteins with different functions. Domains vary in length from ≈25 to 500 amino acids. The shortest domains, such as zinc fingers, are stabilized by metal ions or disulfide bridges. Domains often form functional units, such as the calcium-binding EF-hand domain of calmodulin. Because they are independently stable, domains can be “swapped” by genetic engineering between one protein and another to make chimeric proteins. Identified protein domain functional descriptions were annotated by InterProScan (a sequence analysis application) based on the protein sequence alignment method, and the InterPro domain database was used. InterPro (http://www.ebi.ac.uk/interpro/) is a database that integrates diverse information about protein families, domains, and functional sites and is freely available to the public *via* Web-based interfaces and services. Central to the database are diagnostic models, known as signatures, by which protein sequences can be searched to determine their potential functions. InterPro can be used for large-scale analysis of whole genomes and meta-genomes, as well as in characterizing individual protein sequences.

##### KEGG pathway annotation

KEGG connects known molecular interaction network information such as pathways and complexes (the “Pathway” database), genes and proteins information generated by genome projects (including the gene database) and biochemical compound and reaction information (including compound and reaction databases). These databases are different networks known as “protein network” and “chemical universe.” There are efforts in progress to add knowledge to KEGG, including adding information regarding ortholog clusters to the KEGG Orthology database. KEGG pathways mainly include metabolism, genetic information processing, environmental information processing, cellular processes, rat diseases, and drug development. Kyoto Encyclopedia of Genes and Genomes (KEGG) database was used to annotate protein pathways. First, KEGG database descriptions of proteins were annotated using KEGG online service tools KAAS. Then, KEGG online service tool KEGG mapper was used to map the annotation results to the KEGG pathway database.

##### Subcellular localization

The cells of eukaryotic organisms are elaborately subdivided into functionally distinct membrane-bound compartments. Eukaryotic cells are mainly composed of extracellular space, cytoplasm, nucleus, mitochondria, Golgi apparatus, endoplasmic reticulum (ER), peroxisome, vacuoles, cytoskeleton, nucleoplasm, nucleolus, nuclear matrix, and ribosomes. Bacteria also have subcellular localizations that can be separated when the cell is fractionated. The most common localizations referred to include the cytoplasm, the cytoplasmic membrane (also referred to as the inner membrane in Gram-negative bacteria), the cell wall (which is usually thicker in Gram-positive bacteria), and the extracellular environment. Most Gram-negative bacteria also contain an outer membrane and periplasmic space. Unlike eukaryotes, most bacteria do not contain membrane-bound organelles; however, there are some exceptions. Therefore, we used wolfpsort, a subcellular localization predication software, to predict subcellular localization. Wolfpsort is an updated version of PSORT/PSORT II for the prediction of eukaryotic sequences. Especially for protokaryon species, subcellular localization prediction software CELLO was used.

##### Functional enrichment

###### Analysis of gene ontology enrichment.

Proteins were classified into biological process, cellular compartment, and molecular function according to GO annotations. For each category, a two-tailed Fisher's exact test was employed to test the enrichment of the differentially expressed protein against all identified proteins. The GO with a corrected *p*-value < 0.05 was considered to be significant. Encyclopedia of Genes and Genomes (KEGG) database was used to identify enriched pathways by a two-tailed Fisher's exact test and to test the enrichment of the differentially expressed protein against all identified proteins. The pathway with a corrected *p*-value <0.05 was considered to be significant. These pathways were classified into hierarchical categories according to the KEGG website. InterPro (a resource that provides functional analysis of protein sequences by classifying them into families and predicting the presence of domains and important sites) database was used to perform protein domain enrichment analysis for each category protein, and a two-tailed Fisher's exact test was employed to test the enrichment of the differentially expressed protein against all identified proteins. Protein domains with a corrected *p*-value < 0.05 were considered to be significant.

###### Enrichment-based clustering.

To further hierarchical clustering based on differentially expressed protein functional classification (such as GO, Domain, Pathway, Complex), we first collated all the categories obtained after enrichment along with their *P* values, and then filtered those categories enriched in at least one of the clusters with *P* < 0.05. These filtered *P* value matrixes were transformed by the function *x* = –log 10 (*P* value). Finally, these *x* values were *z*-transformed for each functional category. These *z* scores were then clustered by one-way hierarchical clustering (Euclidean distance, average linkage clustering) in Genesis. Cluster memberships were visualized by a heat map using the “heatmap. 2” function from the “gplots” R-package.

### Statistical analysis

Data analysis was done by SPSS (19.0) computer-based software. Parametric one-way ANOVA with the Tukey test was used to investigate differences between groups (SPSS for Windows, version 11.5, SPSS Inc., Chicago, IL). Values of *P* < 0.05 were considered significant.

## Results

### Chemical composition of AFHM total alkaloids

**Figure 1:**
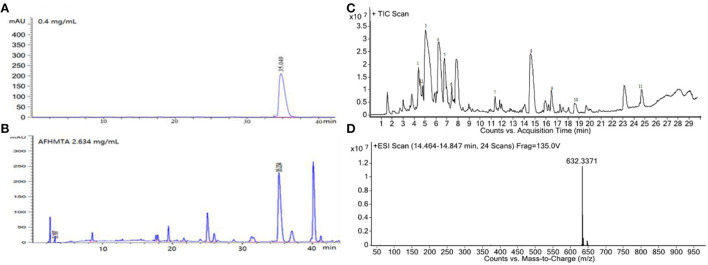
**(A)** HPLC diagram of aconitine standard. **(B)** HPLC diagram of AFHM total alkaloids. **(C)** Q-TOF total ion chromatograms of AFHM total alkaloids; **(D)** Ion m/z diagram of the main component 3-deoxyaconitrile of AFHM total alkaloids.

The AFHM total alkaloids were detected by HPLC/QTOF-MS (Figure 1), and the mass/charge ratio and molecular weight of the detected components were compared with the database of traditional Chinese medicine. There were 11 alkaloids (Table 1), namely 6-O-demethylneoline, songorine, 12-epi-napelline, neoline, 10-hydroxyneoline, jesaconine (25), benzoylaconitin, 3-deoxyaconitine (26), mesaconitine, acetylaconitine, and aconitine (27), which were consistent with existing research reports. The contents of aconitine and 3-deoxyaconitine in total alkaloids were determined by HPLC. The average percentage contents of aconitine and 3-deoxyaconitine in total alkaloids were about 189.63 and 134.32 mg/g.

**Table 1:** Results of Q-TOF tandem mass spectrometry analysis of AFHM total alkaloids.

**Time/min**	**Molecular formula**	**Chemical name**	**m/z**	**Structural formula**
4.393	C_23_H_37_NO_6_	6-O-Demethylneoline	424.2657	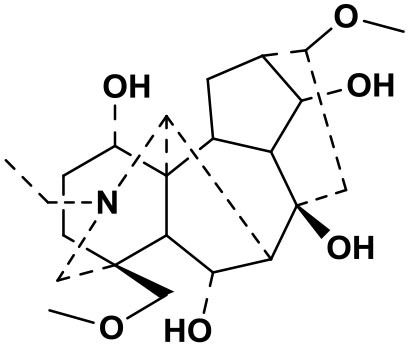
4.743	C_22_H_31_NO_3_	Songorine	358.2347	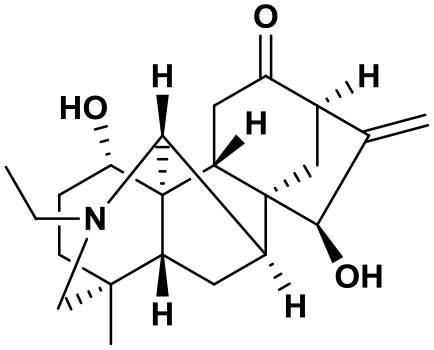
5.427	C_22_H_33_NO_3_	12-epi-Napelline	360.2499	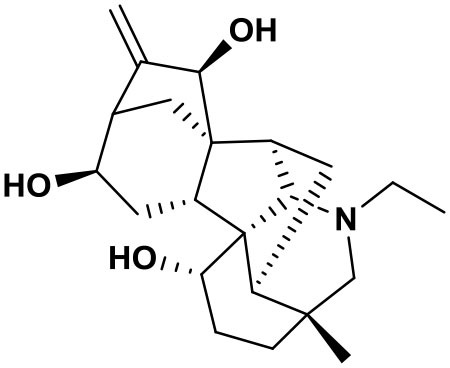
6.461	C_24_H_39_NO_6_	Neoline	438.2807	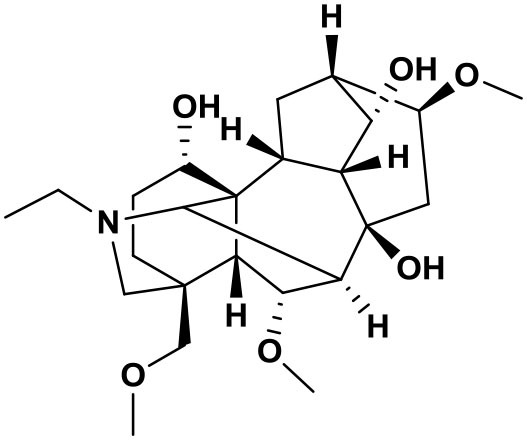
4.743	C_24_H_39_NO_7_	10-Hydroxyneoline	454.2757	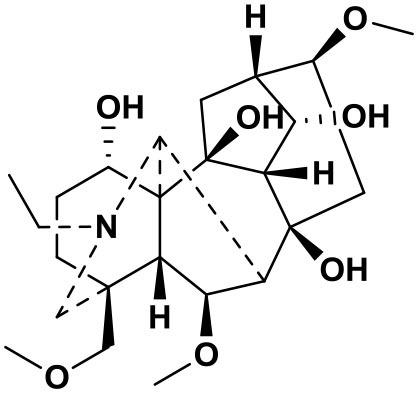
1.559	C_25_H_41_NO_9_	Jesaconine	499.2972	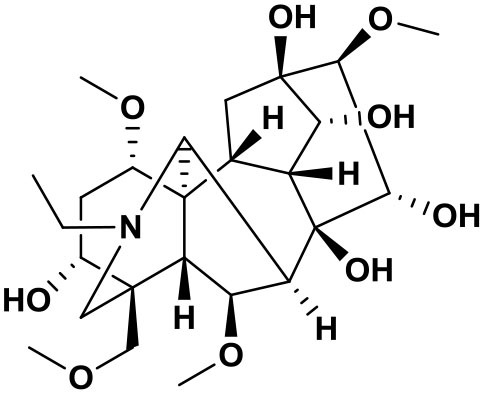
11.329	C_32_H_45_NO_10_	Benzoylaconitin	604.3046	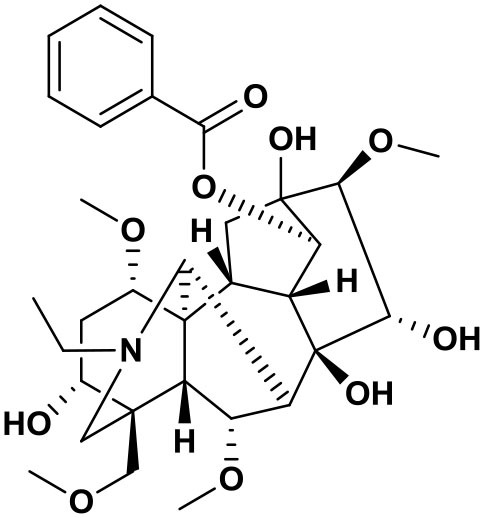
18.598	C_34_H_47_NO_10_	3-Deoxyaconitine	630.2853	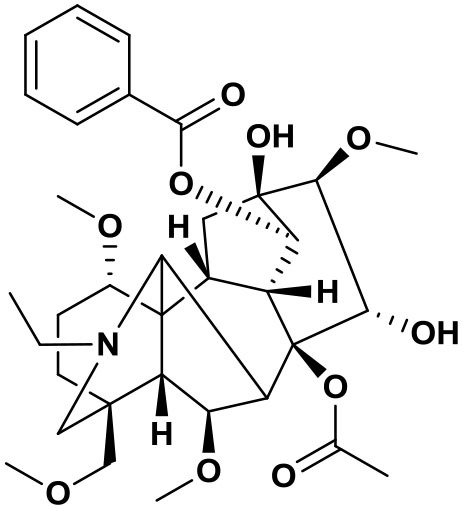
14.847	C_33_H_45_NO_11_	Mesaconitine	632.3371	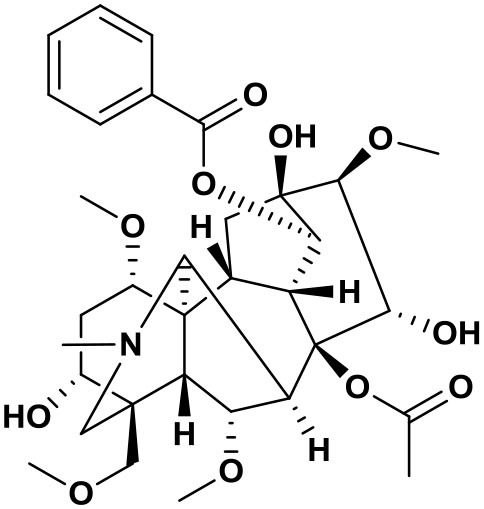
18.598	C_36_H_49_NO_12_	Acetylaconitine	680.3486	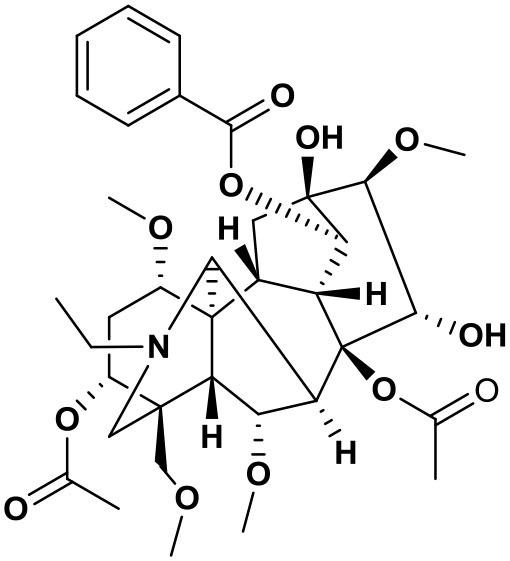
24.634	C_34_H_47_NO_11_	Aconitine	647.4473	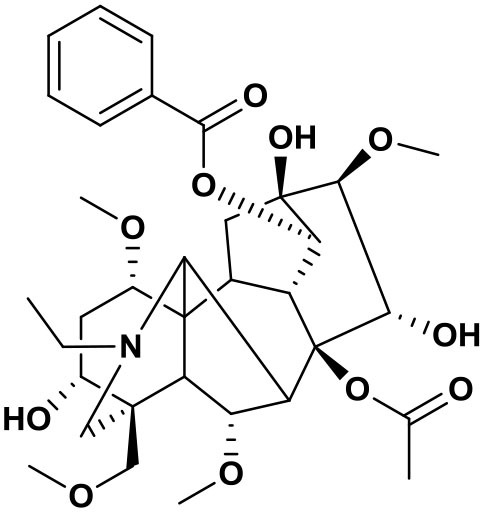

### Killing *melophagus ovinus* activity *in vitro*

**Figure 2:**
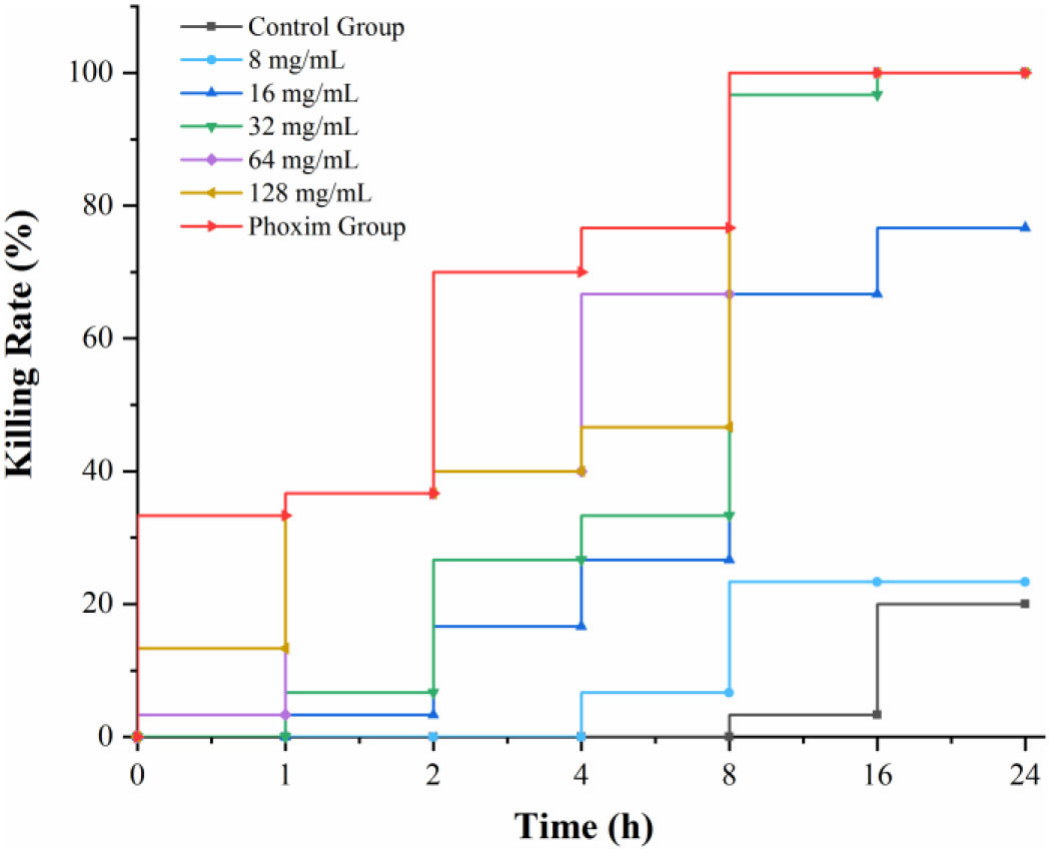
Drug-time action curve of killing *Melophagus ovinus* with AFHM total alkaloids.

According to the statistics of drug-time action, when the concentration of AFHM total alkaloids was 64 mg/ml and the action time was 16 h, the killing rate of *Melophagus ovinus* was 100%, which was the minimum AFHM concentration comparable to the killing rate of the positive control drug (phoxim, the concentration was 1%) in the same action time (Figure 2). In addition, it is generally believed in the industry that the killing rate of antiparasitic drugs within 24 h reaches more than 90% is considered to have a good antiparasitic effect. Therefore, 64 mg/ml of AFHM total alkaloids was considered as the optimal drug concentration and was used for subsequent experiments.

### Differences in body surface of *melophagus ovinus* after drug action

**Figure 3:**
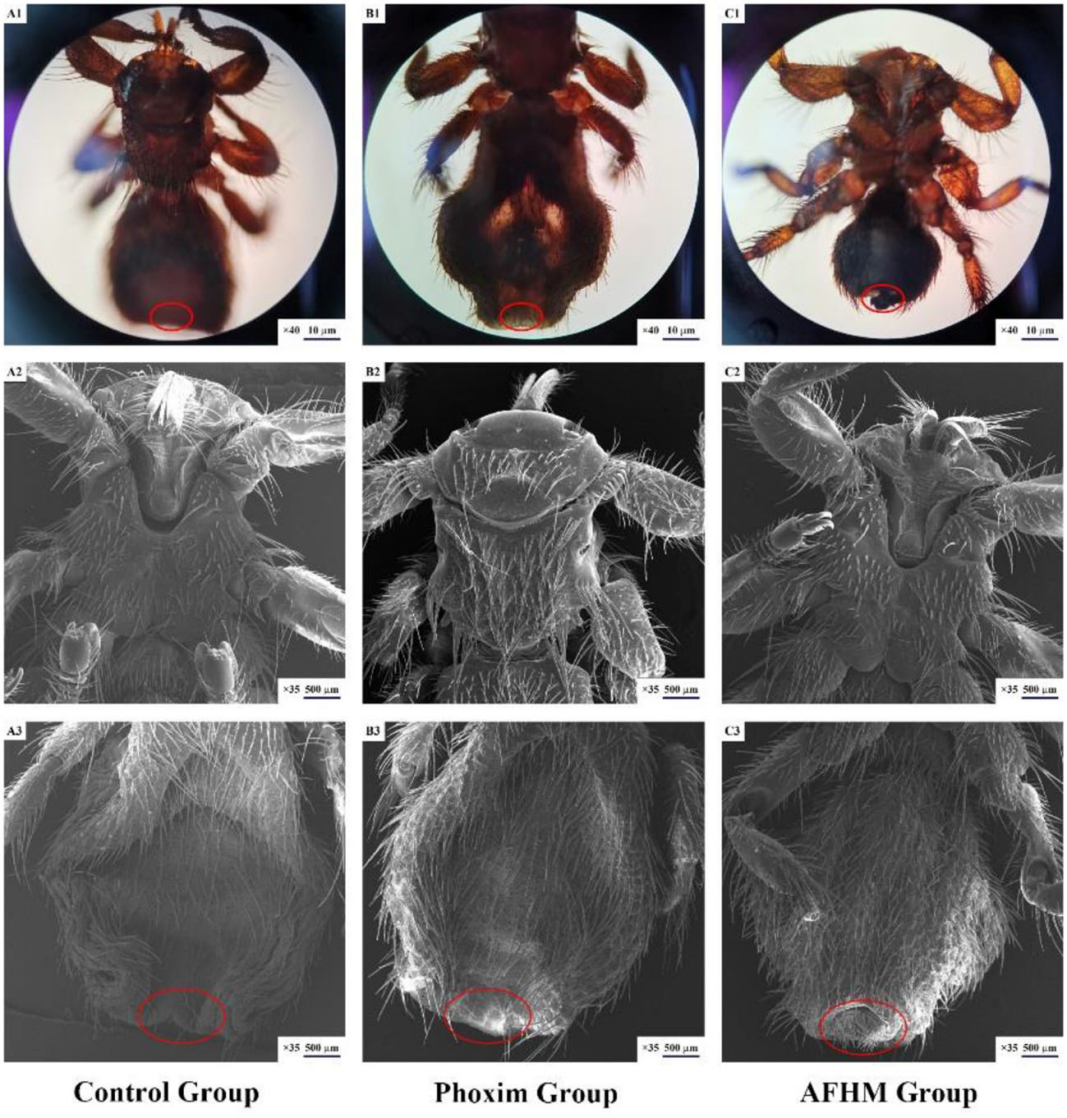
Scanning electron microscopic observation on the body surface of *Melophagus ovinus* after drug action.

Stereomicroscope and scanning electron microscope were used to observe the differences in the body surface of *Melophagus ovinus* after the action of AFHM total alkaloids. The results showed that compared with the blank control group and the positive control group, the morphology of the posterior end of the female *Melophagus ovinus* in the total alkaloid treatment group was significantly different, and most of the epidermal tissue fell off and missing (Figure 3).

### Effect of AFHM total alkaloids on the key enzyme activities of *melophagus ovinus*

**Figure 4:**
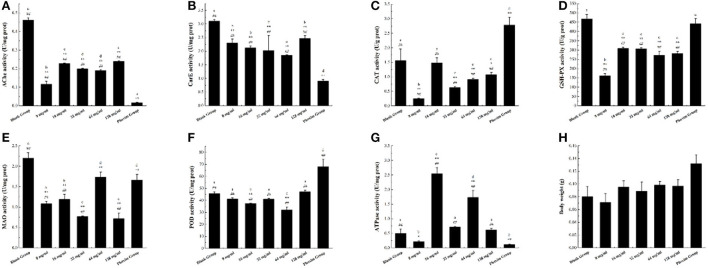
The difference of enzyme activity in *Melophagus ovinus* after treated with AFHM total alkaloids for 16 h. **(A)** Enzyme activity of AchE; **(B)** Enzyme activity of CarE; **(C)** Enzyme activity of CAT; **(D)** Enzyme activity of GSH-Px; **(E)** Enzyme activity of MAO; **(F)** Enzyme activity of POD; **(G)** Enzyme activity of Ca^2+^ Mg^2+^-ATPase; **(H)** Body weight of *melophagus ovinus*. The results were expressed as the mean ± standard deviation of three independent experiments. The results were expressed as the mean ± standard deviation of three independent experiments. *P < 0.05 and **P < 0.01 when compared with the blank control group, ^#^*P* < 0.05 and ^*##*^*P* < 0.01 compared with positive control group was considered statistically significant differences. The significant differences (*P* < 0.05) between groups were indicated by different letters.

Acetylcholinesterase (AChE) catalyzes the degradation of the neurotransmitter acetylcholine, terminates its stimulating effect on the excitation of the postsynaptic membrane, maintains the normal transmission of nerve impulses in organisms, and plays an important role in the regulation of nerve conduction (28). In this study, as shown in Figure 4A, the AchE enzyme activity levels of different concentrations of AFHM total alkaloid groups and the positive control group decreased to varying degrees, indicating that AFHM total alkaloids and phoxim could reduce the AchE enzyme activity and weaken its degradation of acetylcholine. It could cause the transmission of nerve signals in *Melophagus ovinus* to be disturbed, resulting in damage to the nerve cells of *Melophagus ovinus*.

Carboxylesterase (CarE) can catalyze the hydrolysis of endogenous and exogenous substances containing ester bond, amide bond, and thioester bond, participate in lipid transport and metabolism, and is related to the detoxification and metabolism of a variety of drugs, environmental poisons and carcinogens (29). It is one of the important detoxification enzymes in the body of parasites. As shown in Figure 4B, the activity of CarE in each AFHM group was reduced to varying degrees compared with the blank control group after different concentrations of AFHM total alkaloids were acted on *Melophagus ovinus*. Among them, the CarE inhibitory activity of AFHM total alkaloids at 64 mg/ml was significantly better than other concentrations. Studies have demonstrated that organophosphorus pesticides can bind and inhibit the activity of CarE (30). Therefore, it was speculated that AFHM total alkaloids might have a similar binding and inhibitory effect on CarE or effects of affecting lipid transport and metabolism of an organism as positive control drug phoxim.

Under normal growth and metabolism, peroxidase (CAT) greatly improves the maintenance ability of intracellular redox balance of *Melophagus ovinus*. The antioxidant effect of SOD needs the participation and coordination of CAT. Because SOD mainly catalyzes the disproportionation reaction of O^2−^ to produce H_2_O_2_ and O_2_ and eliminates the damage of O^2−^ to cells, and the generated H_2_O_2_ is further decomposed into H_2_O by CAT (31). As shown in Figure 4C, the CAT enzyme activity of *Melophagus ovinus* in the positive control group was significantly higher than that of the blank group. However, the CAT enzyme activities of the AFHM total alkaloid groups at various concentrations were decreased to varying degrees, which indicated that AFHM total alkaloids and the positive control drug phoxim might have different mechanisms of killing *Melophagus ovinus*. Among them, the CAT activity of the 16 mg/ml group was significantly higher than that of the other AFHM total alkaloids concentration groups, suggesting that the “toxic excitation effect (32)” might be generated when total AFHM alkaloids catalyzed the decomposition of H_2_O_2_ at this concentration. That was, high concentrations of AFHM total alkaloids could cause severe oxidative damage in *Melophagus ovinus*, but low concentrations of AFHM total alkaloids (such as 16 mg/ml) might slightly interfere with the homeostasis of *Melophagus ovinus* and initiate a series mechanism of repair and maintenance. For example, low concentrations of AFHM total alkaloids could increase the expression of cytoprotective and repairing proteins by activating transcription factors and kinases, resulting in increased activity of antioxidant enzyme CAT to a certain extent.

GST plays a major role in the detoxification enzyme system *in vivo*. It can change electrophilic compounds into hydrophilic substances by a covalent combination of catalytic substances and their metabolites with the sulfhydryl group of GSH, degrade toxic compounds, and excrete them out of the body (33). GST molecule has the activity of GSH-Px and can repair the function of macromolecules damaged by oxidation, such as DNA and protein. GSH-Px is an important peroxide-decomposition enzyme widely existing in the body. It can reduce toxic peroxides to non-toxic hydroxyl compounds, thereby protecting the structure and function of cell membranes from interference and damage by peroxides. Compared with the blank group, the GSH-Px enzyme activity in *Melophagus ovinus* tissues in the administration group was significantly reduced (*P* < 0.05), as shown in Figure 4D.

MAO is a flavin protein located on the outer membrane of mitochondria, including two subtypes of monoamine oxidase A (MAO-A) and MAO-B. It can catalyze the oxidative deamination of varieties of monoamine neurotransmitters and release hydrogen peroxide at the same time, resulting in cellular oxidative stress (34). Therefore, abnormal MAO activity will lead to the disorder operation of monoamine neurotransmitters in biological cells. In view of the strongest inhibitory effect of AFHM total alkaloids on MAO-B, they were selected as inhibitors to investigate the dose-effect inhibition relationship of MAO-B. The reaction system was prepared at the concentration of 8, 16, 32, 64, and 128 mg/ml respectively, and the system without inhibitors was used as the control. As shown in Figure 4E, the MAO activity of the AFHM total alkaloid group with a concentration of 64 mg/ml was comparable to that of the positive control group, significantly lower than that of the blank control group, but higher than that of other AFHM total alkaloid concentration groups. The reasons for the above results, on the one hand, might be under the action of AFHM total alkaloids, the nervous system damage of *Melophagus ovinus* was aggravated, monoamine neurotransmitters in the body were accumulated, and the demand for oxidative decomposition of MAO was increased, resulting in the inhibition of MAO enzyme activity in experiment groups. On the other hand, nerve cells of *Melophagus ovinus* were damaged by the action of AFHM total alkaloids. The dysfunctional damaged cells put the oxidative decomposition of MAO in an accelerated and violent decomposition dynamic, causing the different levels of MAO enzyme activity under the action of different concentrations of AFHM total alkaloids. Besides, the MAO enzyme activity level of the AFHM total alkaloid group at a concentration of 64 mg/ml was higher than that of other AFHM groups, which further indicated that this concentration was the optimal killing concentration of AFHM total alkaloids for *Melophagus ovinus*. The result was corroborated with the result of the *in vitro* killing experiment of *Melophagus ovinus*.

Protective enzymes can effectively reduce the oxidative damage of cells caused by reactive oxygen species. POD is one of the important protective enzymes involved in the metabolism of reactive oxygen species (ROS), which widely exists in animals, plants, and microorganisms and can effectively reduce the oxidative damage of cells caused by ROS. It directly oxidizes phenolic or amine compounds with H_2_O_2_ as an electron acceptor and has the dual effect of eliminating the toxicity of hydrogen peroxide and phenolic amines. At the same time, it is one of the key factors for pests to resist the oxidative stress of plant secondary substances and form the adaptability of the feeding host. Therefore, to analyze and verify the function of the protective enzyme POD in insect resistance (mite), the changes in POD gene expression and enzyme activity in the host pest should be considered simultaneously (35). As illustrated in Figure 4F, compared with the blank control group, POD enzyme activity in groups with different concentrations of AFHM total alkaloids had no significant difference or decreased to different degrees, while POD enzyme activity in the positive control group was significantly increased. The results indicated that the mechanisms of killing *Melophagus ovinus* between AFHM total alkaloids and the positive control drug phoxim might be different. Among them, the POD enzyme activities of 32 and 128 mg/ml concentrations were increased when compared with other AFHM concentration groups, which might be caused by the increase of oxidative stress and cytotoxic substances in the body of *Melophagus ovinus* under these two drug concentrations.

Ca^2+^Mg^2+^-ATPase is widely distributed in organisms and can catalyze the hydrolysis of ATP molecules to produce ADP molecules and inorganic phosphorus. The activity of the ATP molecular enzyme can be determined by measuring the content of inorganic phosphorus. Ca^2+^-Mg^2+^-ATPase is an important enzyme protein on the cell membrane, which can actively transport Ca^2+^ outside the cell and ingest Mg^2+^. The function of Ca^2+^-Mg^2+^-ATPase is to move Mg^2+^ into the cell and move Ca^2+^out of the cell to maintain the relative stability of the internal environment (36, 37). The AFHM total alkaloids acted on the *Melophagus ovinus*, disrupting its energy metabolism and ion balance, resulting in the dysfunction of the membrane ion pump, the decrease of ATP, and abnormal distribution of ions inside and outside the cell. But at the same time, the effect of AFHM total alkaloids increased the activity of Ca^2+^-Mg^2+^-ATPase, improved the energy metabolism of the body, and protected it from damage (38). As shown in Figure 4G, compared with the blank control group, the activity of Ca^2+^-Mg^2+^-ATPase decreased in the AFHM total alkaloids group with a concentration of 8 mg/ml and the positive control group, but increased in other AFHM total alkaloid concentration groups to varying degrees. Among them, Ca^2+^-Mg^2+^-ATPase activity increased significantly in 16 and 64 mg/ml AFHM concentration groups. It was speculated that under the action of these two concentrations of AFHM total alkaloids, the energy consumption and heat production of Ca^2+^-Mg^2+^-ATPase did not reach 50% of the total heat energy of *Melophagus ovinus* and did not cause disorders of intracellular energy metabolism, ion transport, and signal transduction, resulting little effect on the normal function of *Melophagus ovinus*, but might cause energy consumption damage such as hypoxia in *Melophagus ovinus*. The body weight of *Melophagus ovinus* in each experimental group was shown in Figure 4H, which was used for the description of the body size of *Melophagus ovinus* and the calculation of the extracted total protein content.

### The no reference genome transcriptome sequencing results of *melophagus ovinus*

Through filtering the raw offline data, Reads with connectors, length less than 50 bp and average sequence quality less than were removed (Table 2). The high-quality sequences obtained were spliced ab initio to obtain transcripts. Then the transcripts were clustered, the longest one was selected as Unigene. Finally, the Transcript and Unigene sequences were statistically analyzed and the results were shown in (Table 3). The detailed data of the no reference genome transcriptome sequencing has been uploaded to the NCBI database (PRJNA836644). The basic information for the establishment of sample library was shown in Supplementary Table S1. The statistics of raw data were listed in Supplementary Table S2.

**Table 2:** Data filtering statistics.

**Sample**	**Clean reads no**.	**Clean data (bp)**	**Clean reads %**	**Clean data %**
A	36,564,272	5,484,640,800	93.03	93.03
B	38,719,130	5,807,869,500	93.31	93.31
C	36,551,532	5,482,729,800	90.58	90.58

Sample: sample name; Clean reads No: number of high-quality sequences reads; Clean data (bp): base number of high-quality sequences; Clean reads %: the percentage of high-quality sequence reads in sequencing reads; Clean data %: the percentage of high-quality sequence bases in sequencing bases.

**Table 3:** Overall statistics of sequence splicing results.

**Name**	**Transcript**	**Unigene**
Total Length (bp)	159,105,995	43,731,675
Sequence Number	80,757	35,098
Max. Length (bp)	24,388	24,388
Mean Length (bp)	1970.18	1245.99
N50 (bp)	3,427	2,237
N50 Sequence No.	13,956	5,141
N90 (bp)	822	466
N90 Sequence No.	49,040	23,339
GC%	32.18	32.39

Total length (bp): total length of sequence; Sequence number: total number of sequences; Max. length (bp): maximum length of sequence; Mean length: average length of the sequence; N50 (bp): arrange all sequences from long to short, and add the sequence length in this order. When the added length reaches 50% of the total length of the sequence, the length of the last sequence; N90 (bp): arrange all sequences from long to short, and add the sequence length in this order. When the added length reaches 90% of the total length of the sequence, the length of the last sequence; N50 sequence No.: total number of sequences with length >N50; N90 sequence No.: total number of sequences with length >N90; GC%: GC content of the sequence.

### Protein identification

**Figure 5:**
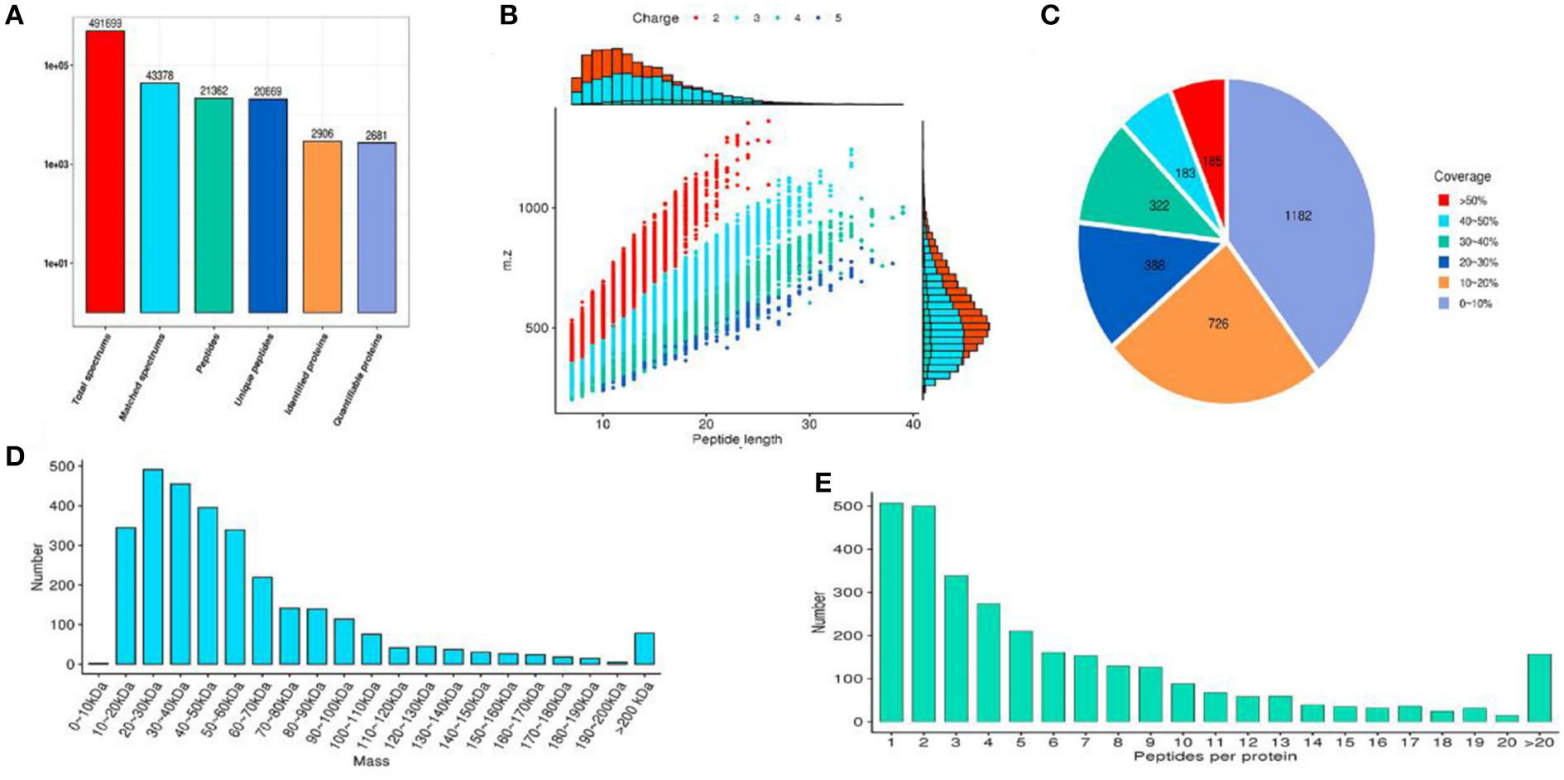
The results of protein identification and data quality control. **(A)** Statistics of protein identified; **(B)** Peptide length distribution; **(C)** Protein coverage distribution; **(D)** Molecular weight distribution; **(E)** Peptide number distribution of proteins.

The identified protein needs to contain at least one unique peptide. We used the TMT-based quantitative proteomic approach to obtain a comprehensive view of the protein changes influenced by irradiation (Table 4). We identified a total of 2,906 proteins, of which 2,681 were quantified (Figure 5). When the *p*-value was < 0.05, a protein fold change >1.2 was considered to indicate a differentially abundant protein (DAP).

**Table 4:** Overview of protein identification.

**Title**	**Number**
Unique peptides	20,669
Total spectrums	491,699
Quantifiable proteins	2,681
Peptides	21,362
Matched spectrums	43,378
Identified proteins	2,906

1. Total spectra: number of total spectra, number of secondary spectra generated by mass spectrometry detection; 2. Matched spectra: number of effective spectra, the number of spectra matched with the theoretical secondary spectra; 3. Peptides: the number of identified peptides, and the number of peptide sequences resolved by the matching results; 4. Unique peptides: the number of identified unique peptides and the number of unique peptide sequences parsed from the matching results; 5. Identified proteins: the number of identified proteins, the number of proteins resolved through specific peptide segments. 6. Quantitative proteins: quantitative protein number, which is the number of proteins quantified through specific peptide segments.

**Figure 6:**
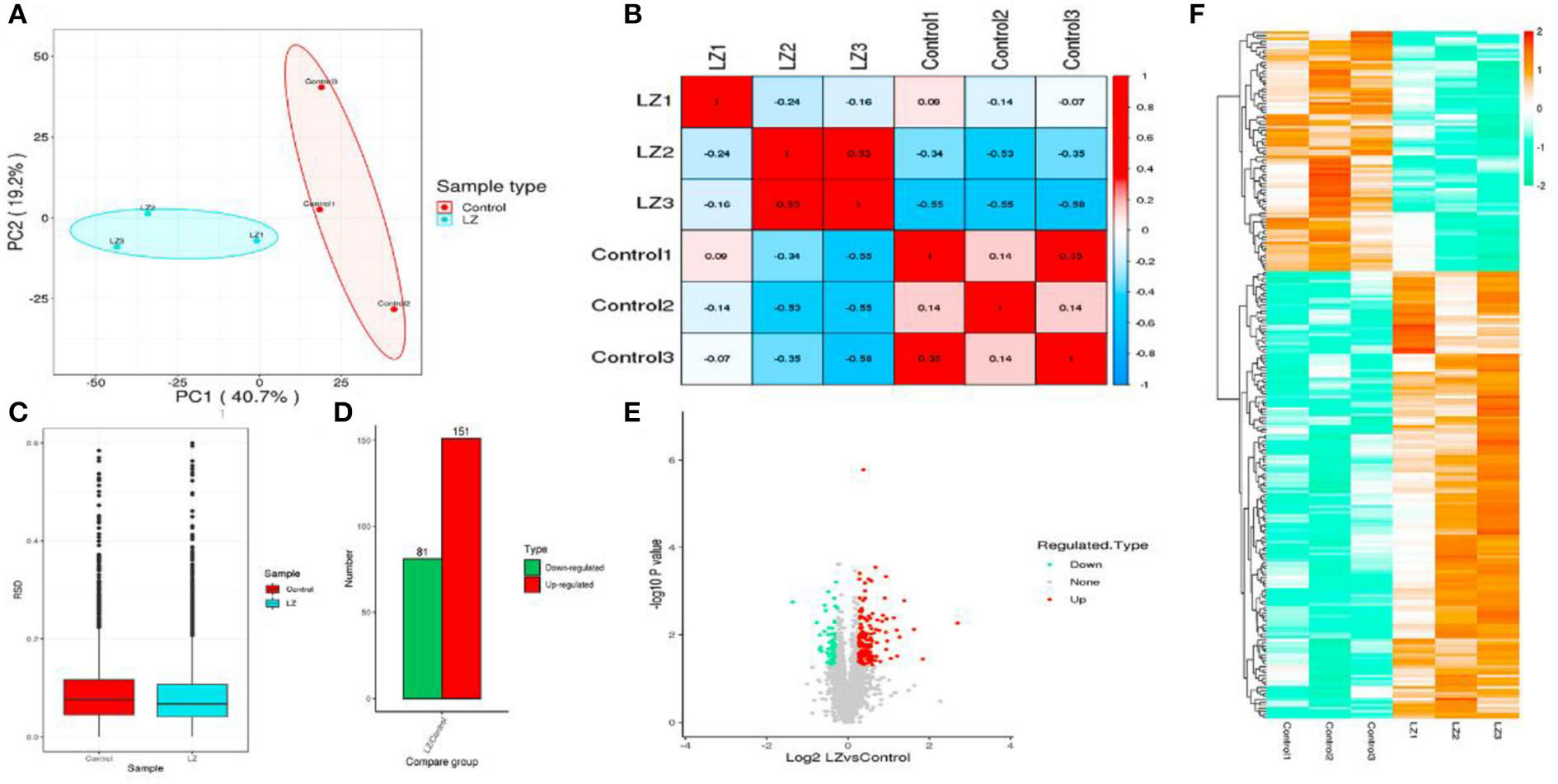
The statistical results of the sample repeatability test and differentially expressed proteins. **(A)** Principal component analysis of quantitative protein; **(B)** Boxplot of relative standard deviation of protein quantitative values between replicates in each group; **(C)** Pearson correlation coefficient between two samples; **(D)** Statistics of differential protein; **(E)** Volcano map of differential proteins; **(F)** Heatmap of differential proteins.

According to the reported intensity of each peptide in different samples given in the library search results, the relative quantitative values of the protein were calculated (Table 5). Three statistical analysis methods, Pearson's correlation coefficient (PCC), principal component analysis (PCA), and relative standard deviation (RSD), were used to evaluate the repeatability of the sample. When *p* value < 0.05, the threshold of change for significant up-regulation was >1.2 and <1/1.2. Finally, we detected 151 upregulated and 81 downregulated proteins (Figure 6).

**Table 5:** Screening results of differential proteins.

**Compared sample name**	**Up regulated**	**Down regulated**
LZ/Control	151	81

### Annotation analysis of the differential proteins

The three categories (biological process, cellular component, and molecular function) in GO classification were enriched and analyzed, respectively. The subcellular structure of proteins was annotated by the PSORTb (V3.0) software, and COG/KOG function classification statistics were carried out.

**Figure 7:**
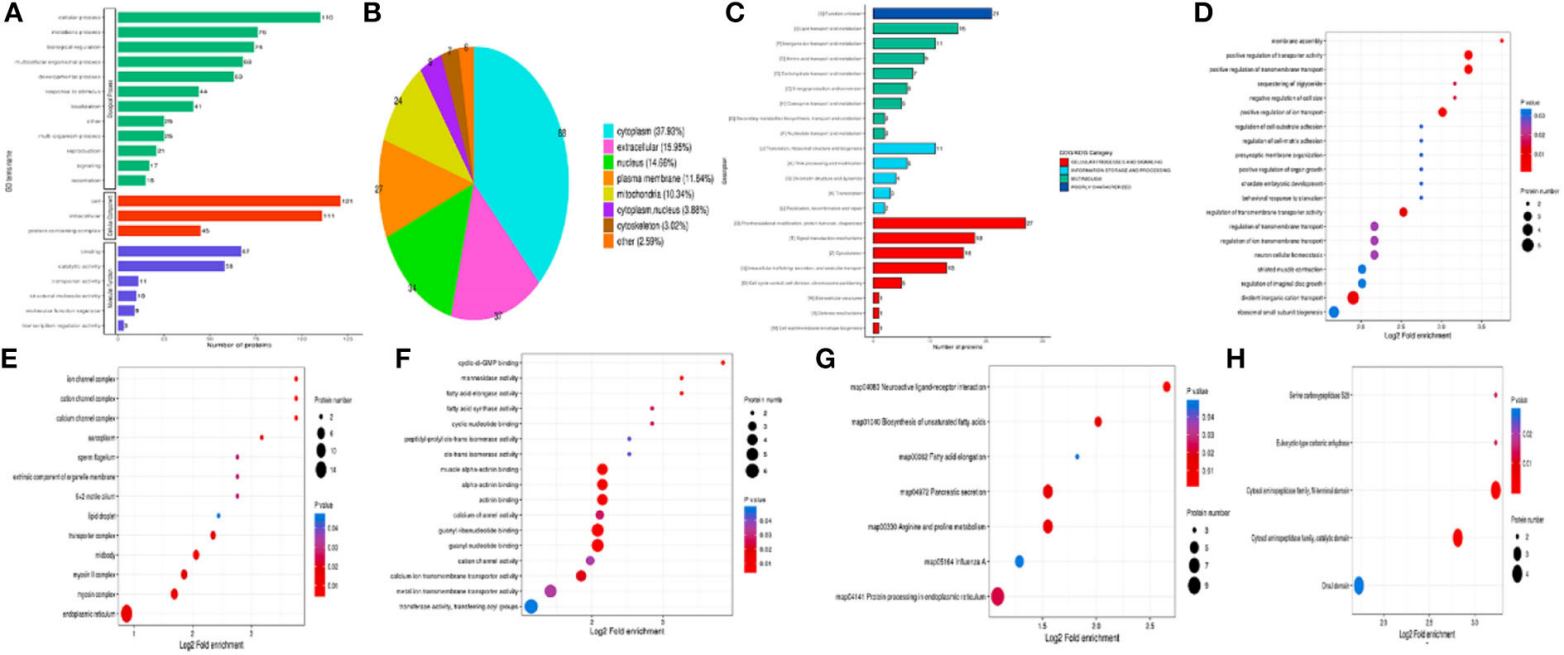
Annotation analysis of the differential proteins. **(A)** GO secondary annotation classification diagram of differential proteins; **(B)** Subcellular structure annotation classification map of differential proteins; **(C)** COG/KOG functional classification map of differential proteins; **(D)** Enrichment analysis of biological process; **(E)** Enrichment analysis of cellular component; **(F)** Enrichment analysis of molecular function; **(G)** Enrichment analysis of KEGG pathway; **(H)** Enrichment analysis of protein domain.

The differentially expressed proteins in the comparison group were enriched at three levels of GO classification, KEGG pathway, and protein domain (Figure 7, Fisher's exact test). It was found that the differential proteins number of KEGG pathway enrichment was up to 10, and the differentially expressed proteins had a significant enrichment trend during protein processing in the endoplasmic reticulum.

**Figure 8:**
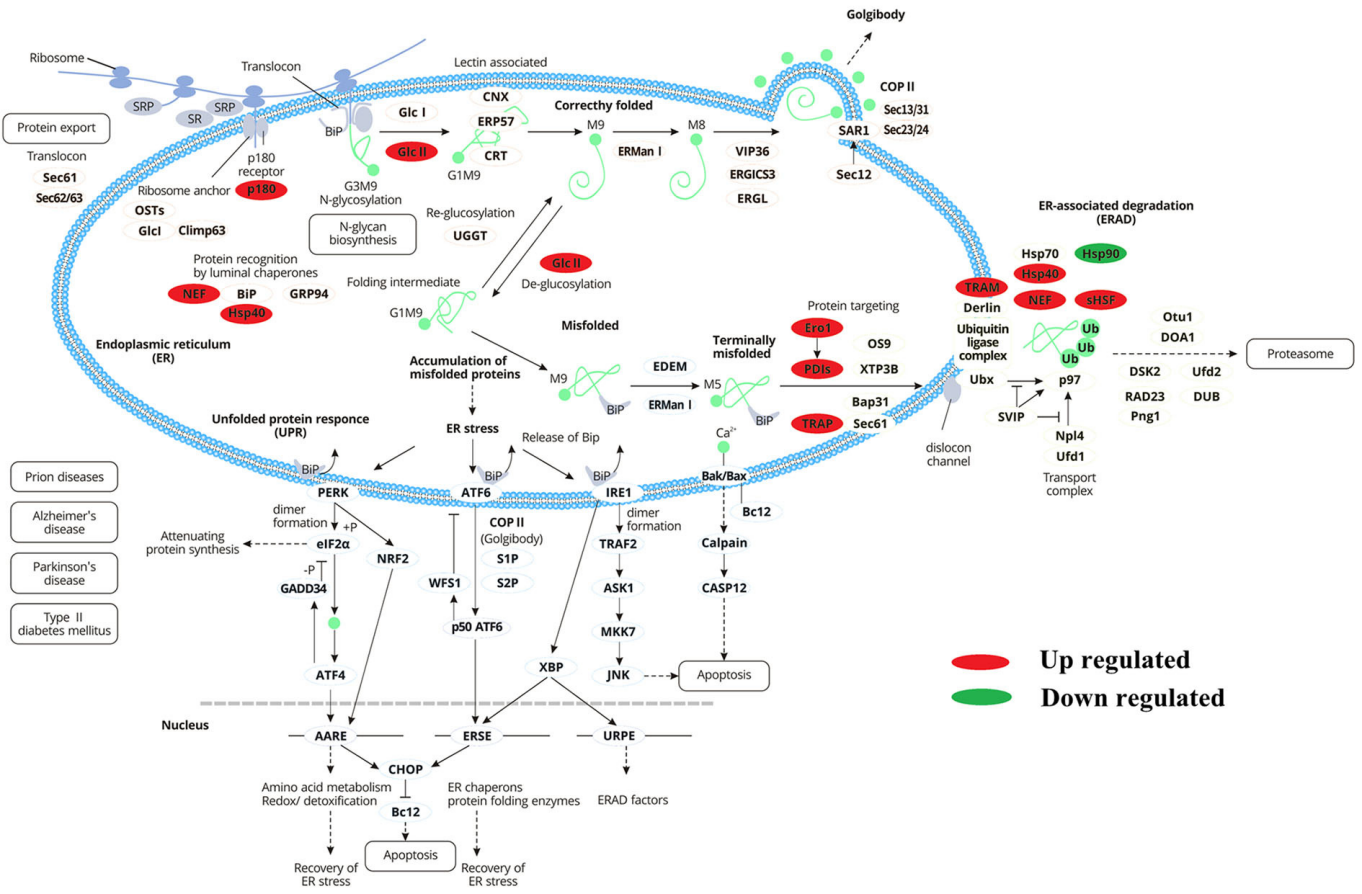
Differentially expressed proteins enrichment trend during endoplasmic reticulum protein processing.

Nine proteins were up-regulated (P180, Hsp40, GlcII, Ero I, PDIs, TRAM, TRAP, Nef, and sHSF) during protein processing in the endoplasmic reticulum, and one protein (Hsp90) was down-regulated (Figure 8). It was speculated that the AFHM total alkaloids may cause the imbalance of PDIs protein expression by affecting the regulation of protein homeostasis of Hsp40 cells and the oxidation of PDI isomerase and related proteins. This will affect the selective recognition of signal sequence, the targeted transport of Sec 61, the correct folding of the three-dimensional structure of amino acid chain, weakened the clearance of amino acid chains that cannot be folded correctly due to damage and the escort of protein molecules to the target molecules, thereby resulting in the killing of *Melophagus ovinus*.

### Cluster analysis

**Figure 9:**
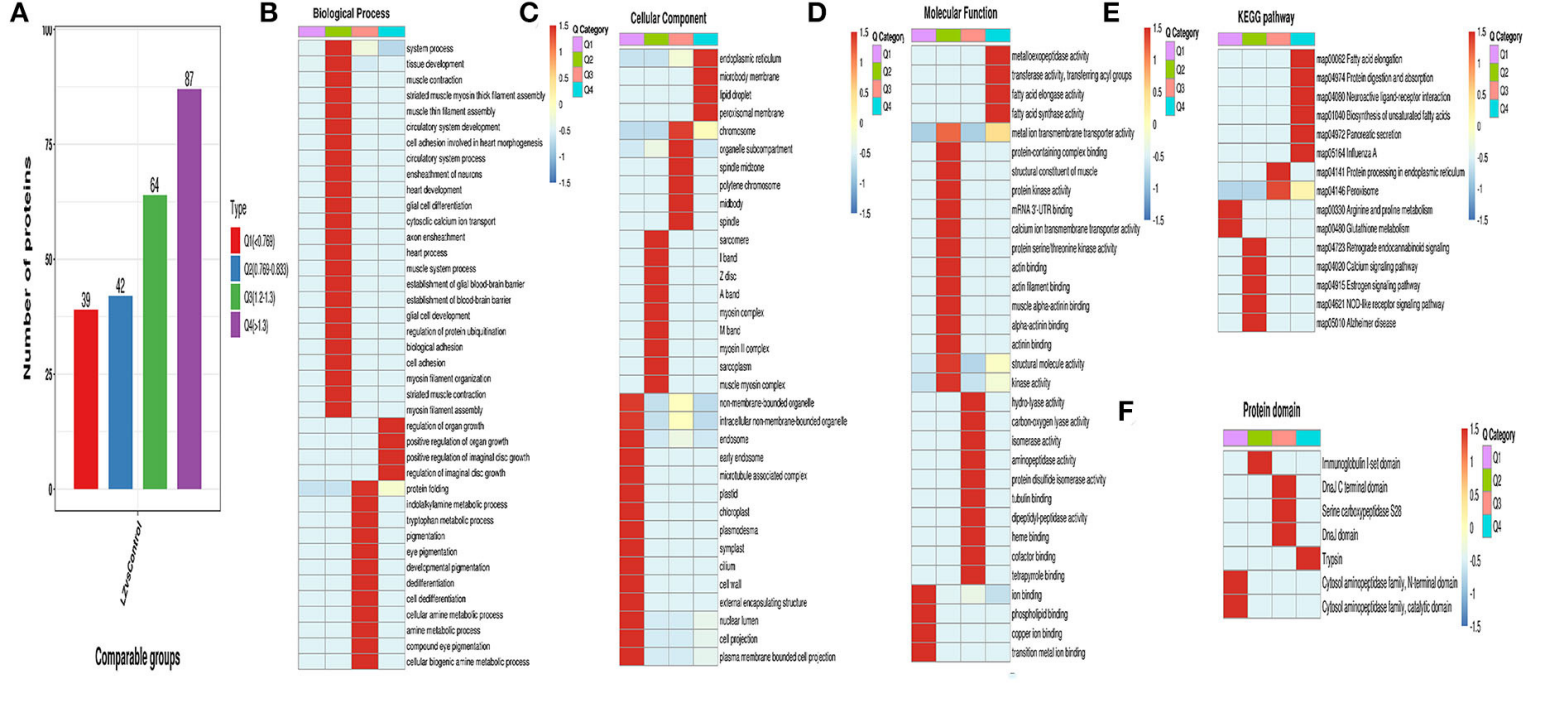
Cluster analysis of differentially expressed proteins. **(A)** Classification diagram of differentially expressed proteins; **(B)** Functional enrichment heatmap of biological process in differential expression protein; **(C)** Functional enrichment heatmap of cellular component in differential expression protein; **(D)** Functional enrichment heatmap of KEGG pathway in differential expression protein; **(F)** Functional enrichment heatmap of protein domain in differential expression protein.

The differentially expressed proteins in the comparison group were classified into GO, KEGG pathway, and protein domain enrichment, and they were further divided into four parts according to the differential expression multiple, called Q1–Q4, as shown in Figure 9A. Then, GO classification, KEGG pathway, and protein domain enrichment were carried out for each Q group, and cluster analysis was performed to find the correlation of protein functions and different differential expression multiples in the comparison group (Figures 9B–F).

### Protein-protein interaction network

**Figure 10:**
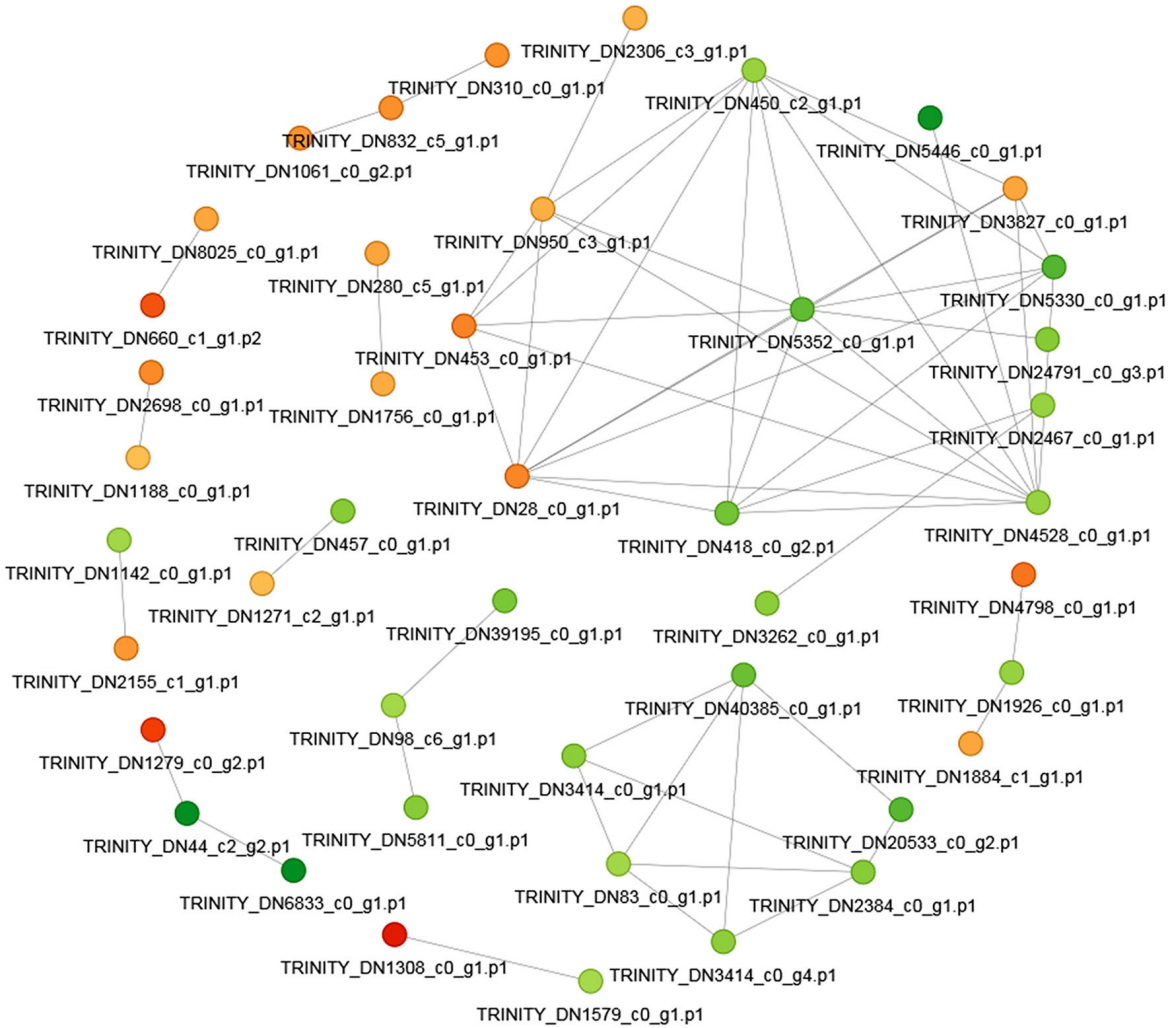
The protein-protein interaction network. The circle in the figure represents the differential protein. Different colors represent the differential expression of the protein (green is the down-regulated protein and red is the up-regulated protein). The darker the color, the greater the difference multiple.

In order to clearly show the interaction relationship between different proteins, the top 50 proteins with the closest interaction relationship were selected to draw the protein-protein interaction network (Figure 10). Among the top 50 differentially expressed proteins, 20 proteins were up-regulated and 30 proteins were down-regulated.

## Discussion

In the process of separation, since AFHM contains a large amount of diterpenoid alkaloids, the separation effect of ordinary column chromatography was not ideal, we mainly used normal-phase silica gel chromatography and reverse-phase C18 chromatography for separation (39). Q-TOF and gel chromatography were used to separate the compounds with good point formation, and 11 alkaloids were finally obtained.

*Melophagus ovinus*, also termed the sheep ked, is a kind of wingless fly that belongs to the order Diptera, family Hippoboscidae. *Melophagus ovinus* is one of the most common hematophagous ectoparasites of sheep, mainly distributed in the neck, shoulder, perineum, and hind legs of animals (40). The life cycle of *Melophagus ovinus* includes four developmental stages: larva, pupa, nymph, and adult, all of which occur on the wool of the host (41). Although sheep are generally considered to be a definitive host of this ectoparasite, *Melophagus ovinus* can also parasitize the body surfaces of goats (42), European bison (43), rabbits and humans (5), and red foxes (33, 44). Furthermore, *Melophagus ovinus* may transmit genus bacteria of the Bartonella, parasites (such as trypanosomes) (9, 45–47), which also threaten the animals' health (48). Nelson et al. (49) stated that long-wool breeds appeared to be particularly susceptible. Although sheep are generally considered to be the only definitive host, reports of *M. ovinus* infecting a range of domesticated and wild animals, including goat (8), *Bison bonasus, Oryctolagus cuniculus*, dog, human, and Vulpes (10) are not uncommon.

Traditional Chinese medicine, especially botanical and animal medicine, are living entities whose characteristics, such as external morphology, color, size, texture, characteristic odor of cross-section, and other microscopic characteristics of chemical composition, are largely determined by the genetic code-gene of the medicinal material. Of course, we cannot ignore the influence of environmental factors. Although genes are the source of genetic information, functional proteins are the executors of gene functions (50, 51). The realization of the genome project has laid a solid foundation for the determination of the entire gene sequence of biological organisms and future life science research (52). However, it cannot provide a direct molecular basis for understanding various life activities, which requires the study of the important link of the executor of life activities—protein (53).

Proteomics technology is currently used to study diseases with complex pathogenesis, such as diabetes, hypertension, and metabolic syndrome. The term proteome was originally coined by Australian scientists Wilkins and Williams in 1994 and was later defined as all proteins expressed by a genome, cell, or cell culture under specific conditions. Proteomics is the science of analyzing and describing biological proteins, protein-protein interactions, and protein modifications to systematically determine the expression of each protein in a cell or tissue, the composition of protein complexes, their interrelationships, and their abundance and modification status. In this experiment, a proteomic study of the killing effect of the AFHM total alkaloids on *Melophagus ovinus* was carried out. The protein extraction and bioinformatics analysis of the drug group and the control group showed that there were significant differences in the expression of some proteins, 80 proteins were up-regulated, 57 proteins were down-regulated, and 44.53% of the differential proteins existed in the cytoplasm. Some proteins that mainly exist in cells, organelles, organelle membranes, and other cellular compositions have significant differences in expression. Most of these proteins are involved in organic matter metabolism, cell metabolism, biological process regulation, primary metabolic process, nitrogen compound metabolism, anatomical structure development, and other biological processes, their main molecular functions include binding proteins, binding organic cyclic compounds, binding heterocyclic compounds, binding ions, hydrolase activity, etc. In this study, the TMT differential proteomics research method was adopted, and all differential proteins were taken as the research objects. by searching and evaluating through the retrieval and evaluation of the differential proteins, the molecular evolution relationship network of the interaction between the drugs and *Melophagus ovinus* was constructed. Furthermore, the mechanism of the effective parts of AFHM against *Melophagus ovinus* was discussed.

## Conclusion

In this paper, HPLC/QTOF-MS was used to detect the constituents of alkaloids extracted. Eleven compounds in the comparison database were consistent with those reported in the literatures. Aconitine was determined by HPLC, and the content was 1.2195 mg/g. About 64 mg/ml of AFHM total alkaloids could achieve a 100% insecticidal rate within 16 h, which was in line with the industry's evaluation standard of good anti-parasite effect. This concentration was the minimum AFHM total alkaloids concentration equivalent to the killing rate of positive control drug phoxim to *Melophagus ovinus in vitro* during the same action time. Therefore, 64 mg/ml of AFHM total alkaloids was considered as the optimal drug concentration and was used for subsequent experiments. When the action time was 16 h and the total alkaloids concentration of AFHM was 64 mg/ml, the activity of Ca^2+^-Mg^2+^-ATPase increased significantly when compared with the blank control group (*P* < 0.01), while AChe, CarE, CAT, GSH-Px, MAO, and POD enzyme activities were significantly decreased (*P* < 0.01). Protein extraction and bioinformatics analysis of the total alkaloid treated group and control group showed that 80 proteins were up-regulated and 57 proteins were down-regulated. The COG/KOG classification mainly played the roles of post-translational modification, protein turnover and chaperone, signal transduction, intracellular transport, secretion and vesicular transport, cytoskeleton constitution, and other functions. The GO enrichment and distribution bubble diagram showed that the differential proteins related to the regulation of RNA biosynthesis, nucleic acid regulation template transcription, transcription regulation DNA template, and other biological processes were significantly different (*P* < 0.01). Some differential proteins were involved in the metabolic pathways of thiazoline-6-mercaptopurine, some affected the basic transcription factors of eukaryotic cells, and some might influence the cell phagocytosis process and promote the production or action pathways of ATPase. This study obtained the comprehensive effect of AFHM total alkaloids on *Melophagus ovinus* body proteins by using TMT technology, enzyme activity detection, and bioinformatics analysis, screening out potential drug-protein targets, providing a theoretical basis for elucidating the mechanism of AFHM killing *Melophagus ovinus*.

## Data availability statement

The datasets presented in this study can be found in online repositories. The names of the repository/repositories and accession number(s) can be found below: https://www.ncbi.nlm.nih.gov/, bioproject/836644.

## Author contributions

XiW, BH, and SW conceived and designed the study. BH and SW conducted the experiments, XiW, ZY, and BH drafted the manuscript. XiW, YZ, FC, XX, FW, YH, CC, BW, PB, XuW, YL, HZ, and BH supervised the study. XiW, ZY, YZ, BH, and SW reviewed the methods and the results. All authors read and approved the manuscript.

## Funding

This work was financially supported by the Central Public-interest Scientific Institution Basel Research Fund (No. 1610322020008) and the Innovation Program of Chinese Academy of Agricultural Sciences – Veterinary Natural Medicine and Antibiotic Replacement (No. 25-LZIHPS-03). Thanks for the technical support provided by PTM Biolabs Inc (Hangzhou) and Shanghai Personal Biotechnology Co., Ltd.

## Conflict of interest

The authors declare that the research was conducted in the absence of any commercial or financial relationships that could be construed as a potential conflict of interest.

## Publisher's note

All claims expressed in this article are solely those of the authors and do not necessarily represent those of their affiliated organizations, or those of the publisher, the editors and the reviewers. Any product that may be evaluated in this article, or claim that may be made by its manufacturer, is not guaranteed or endorsed by the publisher.
