# Supplementary material for: Tandem mass tag labeled quantitative proteomic analysis of differential protein expression on total alkaloid of Aconitum flavum Hand.-Mazz. against melophagus ovinus
Source: Front Vet Sci. 2022 Jul 27;9:951058. doi: 10.3389/fvets.2022.951058 (PMC9365070; doi:10.3389/fvets.2022.951058)
Supplement: Supplementary file 1 [file Table_1.DOCX]

**Supplementary Table S1.** Basic information of Library

| **Sample** | **Lib. Name** | **Lib. Insert Size** | **Sequencing Platform** | **Sequencing Mode** |
| --- | --- | --- | --- | --- |
| A | LRA55740 | 380 bp | Illumina | Paired-end, 2×150 bp |
| B | LRA55741 | 380 bp | Illumina | Paired-end, 2×150 bp |
| C | LRA55742 | 380 bp | Illumina | Paired-end, 2×150 bp |
