# Supplementary material for: Tandem mass tag labeled quantitative proteomic analysis of differential protein expression on total alkaloid of Aconitum flavum Hand.-Mazz. against melophagus ovinus
Source: Front Vet Sci. 2022 Jul 27;9:951058. doi: 10.3389/fvets.2022.951058 (PMC9365070; doi:10.3389/fvets.2022.951058)
Supplement: Supplementary file 2 [file Table_2.DOCX]

**Supplementary Table S2.** Statistics of raw data

| **Sample** | **Reads No.** | **Bases (bp)** | **Q30 (bp)** | **N (%)** | **Q20 (%)** | **Q30 (%)** |
| --- | --- | --- | --- | --- | --- | --- |
| A | 39301828 | 5895274200 | 5489063521 | 0.002545 | 97.59 | 93.1 |
| B | 41493032 | 6223954800 | 5786331812 | 0.002537 | 97.53 | 92.96 |
| C | 40348810 | 6052321500 | 5640361968 | 0.002536 | 97.61 | 93.19 |

**Note:** sample: sample name; Reads No.: total number of reads; Bases (BP): total number of bases; Q30 (BP): the total number of bases whose base recognition accuracy is more than 99.9%; N (%): percentage of fuzzy bases; Q20 (%): percentage of bases with base recognition accuracy of more than 99%; Q30 (%): the percentage of bases with base recognition accuracy of more than 99.9%.
